# Fecal Viral Load and Norovirus-associated Gastroenteritis

**DOI:** 10.3201/eid1208.060081

**Published:** 2006-08

**Authors:** Martin C.W. Chan, Joseph J.Y. Sung, Rebecca K.Y. Lam, Paul K.S. Chan, Nelson L.S. Lee, Raymond W.M. Lai, Wai K. Leung

**Affiliations:** * The Chinese University of Hong Kong, Shatin, Hong Kong Special Administrative Region, People's Republic of China

**Keywords:** Norovirus, viral load, gastroenteritis, dispatch

## Abstract

We report the median cDNA viral load of norovirus genogroup II is >100-fold higher than that of genogroup I in the fecal specimens of patients with norovirus-associated gastroenteritis. We speculate that increased cDNA viral load accounts for the higher transmissibility of genogroup II strains through the fecal-oral route.

Norovirus (NoV), a member of the family *Caliciviridae* in the genus *Norovirus*, is a major causative agent of viral gastroenteritis, affecting all age groups worldwide (*1*). NoVs are clustered into 5 genogroups; genogroup I (GI), GII, and GIV infect humans (*2*). Molecular epidemiologic studies in different countries and regions show that NoV GII is the predominant genogroup circulating in the community; it accounts for most sporadic, nosocomial, and outbreak cases (*3*). However, its predominance cannot be entirely explained. In this study, we show for the first time that the median cDNA viral load of NoV GII is >100-fold higher than that of GI in fecal specimens of patients with NoV-associated gastroenteritis. This finding suggests possible higher transmissibility of GII strains through the fecal-oral route.

## The Study

From December 2004 through November 2005, a total of 651 fecal specimens were collected within 48 hours of symptom onset from 627 patients (43.5% male, <1–97 years of age, 26.9% <16 years of age) with symptoms of gastroenteritis at Prince of Wales Hospital, Hong Kong Special Administrative Region, People's Republic of China. All cases were sporadic (defined as having no known related cases). Fecal specimens were stored at -70°C after collection and were processed in batches monthly. Local monthly mean air temperature during the study period was obtained from the Hong Kong Observatory (available from http://www.hko.gov.hk/wxinfo/pastwx/ywx.htm).

Viral RNA was purified from fecal specimens and transcribed to cDNA as described (*4*). All specimens had a detectable level of human β-actin cDNA, which suggests high RNA integrity. Filter tips were used throughout the study to minimize cross-contamination. NoV GI and GII were detected by a quantitative and genogroup-specific real-time PCR assay, as previously described (*5*). Sterile water was used in place of cDNA as negative control. Three amplicons from each genogroup were directly sequenced to confirm their identities and genogroups on a 3100 Genetic Analyzer (Applied Biosystems, Foster City, CA, USA). cDNA viral load was quantified in triplicate per run against 10-fold serial dilutions (10^8^–10^1^ copies) of external plasmid standards prepared by cloning genogroup-specific amplicons into vector pCR2.1-TOPO (Invitrogen Corp., Carlsbad, CA, USA). The lower detection limit of the assay was equivalent to 2×10^4^ copies of cDNA per gram of fecal specimen. Coefficients of variation within and between runs were calculated as the percentage of the ratio between the standard deviation and mean of threshold cycle numbers from the standard curves. The respective intra- and interassay coefficients of variation for NoV GI were 0%–4.1% and 1.9%–5.8%, respectively, and 0.1%–6.1% and 2.7%–6.6%, respectively, for NoV GII. These findings indicate the high reproducibility in viral load quantitation by the assay. Two common gastroenteritis-associated viruses, sapovirus and group A rotavirus, were detected in parallel, as previously described (*4**,**6*).

Phylogenetic analysis of NoV isolates was performed by using primer sets G1FF/G1SKR and G2FB/G2SKR for GI and GII, respectively, as described elsewhere (*7*). Isolates were clustered with the nomenclature system of Zheng et al. (*2*). Since G1FF/G1SKR and G2FB/G2SKR completely spanned the region amplified by the RT-PCR assay, the 2 primer sets were also used to check for sequence complementarity among target, primers, and probe used in quantitation. Statistical analyses were performed by SPSS version 11.5.1 (SPSS Inc., Chicago, IL, USA), and figures were constructed by Prism version 4.03 (GraphPad Software, Inc., San Diego, CA, USA) and SPSS.

NoVs were detected in 54 (8.3%) fecal specimens. Among the NoV-positive specimens, 8 (14.8%) were infected with GI, 37 (68.5%) with GII, and 9 (16.7%) were coinfected with GI and GII. Moreover, 3 (5.6%) specimens were coinfected with sapovirus, 2 (3.7%) with group A rotavirus, and 1 (1.9%) with sapovirus and group A rotavirus. The mean age of patients infected with NoV GI and GII was 54.6 and 33.0 years, respectively (p = 0.02). Sex and hospitalization rates between patients infected with the 2 genogroups did not differ significantly (Table).

**Table:** Characteristics of patients infected with norovirus (NoV) genogroup I (GI) and GII

Characteristics	GI (n = 8)	GII (n = 37)	GI/II coinfection (n = 9)	p value*
Male sex, no. (%)	3 (38)	21 (57)	4 (44)	0.44
Mean age, y (range)	54.6 (13–85)	33.0 (1–74)	42.2 (12–65)	0.02
Hospitalization, no. (%)	4 (50)	11 (30)	1 (11)	0.41

*NoV GI versus GII only.

**Figure 1:**
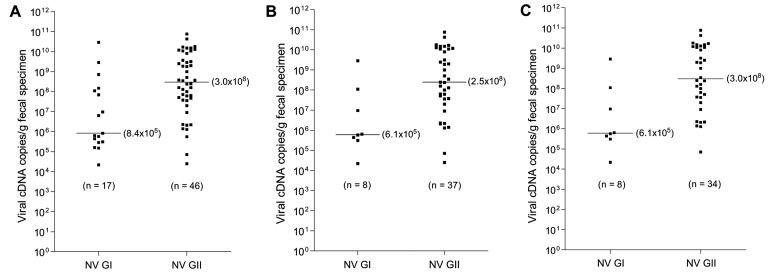
Scatterplots for cDNA viral load of noroviruses (NoV) genogroup I (GI) and GII. A) All positive isolates. B) All positive isolates, excluding those with NoV GI/GII coinfection. C) All positive isolates, excluding all those with viral coinfection (NoV GI and GII together with sapovirus, group A rotavirus, or both). The bars represent median cDNA viral loads. The p values were calculated by 2-tailed Mann-Whitney U test.

**Figure 2:**
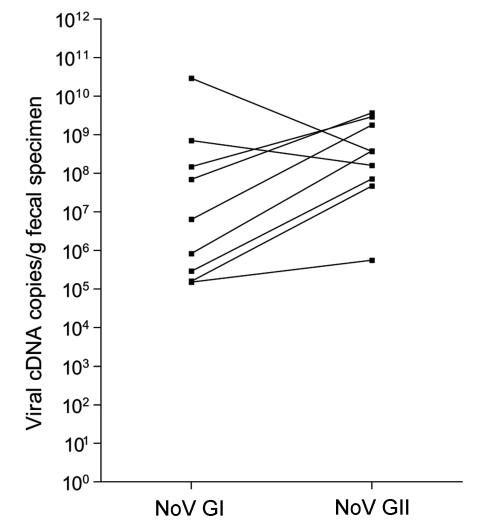
cDNA viral load in 9 specimens with norovirus (NoV) genogroup I (GI) and GII coinfection.

**Figure A1:**
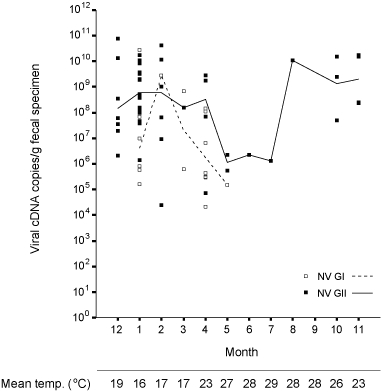
Monthly distribution of cDNA viral load of norovirus (NoV) genogroup I (GI) and GII during the study period (December 2004 through November 2005). In the scatterplot, cDNA viral loads of NoV GI and GII are represented by open and solid squares, respectively. Lowess fit lines for the seasonal variation of the cDNA viral loads of NoV GI and GII are represented by dashed and solid lines, respectively. Local monthly mean air temperature (temp.) is tabulated.

The median cDNA viral load of NoV GI and GII detected in the fecal specimens was 8.4×10^5^ (range 2.2×10^4^–2.9×10^10^) and 3.0×10^8^ (range 2.5×10^4^–7.7×10^10^) copies per gram of fecal specimen (Figure 1A), respectively. Although the range was comparable between the genogroups, the median of NoV GII was >100-fold higher than that of GI (p = 0.0022, 2-tailed Mann-Whitney U test). Similar findings were obtained when NoV GI/GII coinfections (p = 0.0066, Figure 1B) or all viral coinfections (p = 0.0042, Figure 1C) were excluded. Seven of 9 specimens with NoV GI/GII coinfections had higher cDNA viral load of GII than GI, with fold changes from 4 to 452 (median 248) (Figure 2). Furthermore, while NoV was detected year-round, a marked seasonal trend was evident: higher prevalence occurred in winter months, when the cDNA viral load of GII was generally higher compared with GI (Figure A1).

Multivariate linear regression model was used to determine the potential association between cDNA viral load, NoV genogroup, and patient's age. After age stratification, the cDNA viral load in fecal specimen was still significantly associated with NoV genogroup (β = 0.390, p = 0.002). However, no significant association was found between cDNA viral load and age of patients (β = -0.060, p = 0.626).

**Figure A2:**
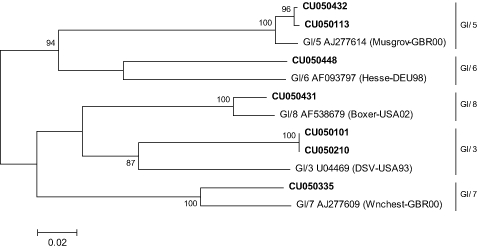
Phylogenetic analysis of norovirus genogroup I nucleotide sequences. The phylogenetic tree was generated by neighbor-joining analysis of the partial nucleotide sequences of the capsid N-terminal/Shell domain with bootstrapping of 1,000 replicates using MEGA version 3.1. Isolates identified in this study are highlighted in **boldface**. Bootstrap values >80 are shown at each node as percentage of 1,000 replicates. The scale bar represents the estimated number of substitutions per site. Nucleotide sequences obtained in this study were deposited in GenBank under accession nos. DQ522116–DQ522122. Nucleotide sequences of reference strains: DSV-USA93 (GenBank accession no. U04469, genotype GI/3), Musgrov-GBR00 (AJ277614, GI/5), Hesse-DEU98 (AF093797, GI/6), Wnchest-GBR00 (AJ277609, GI/7), and Boxer-USA02 (AF538679, GI/8).

**Figure A3:**
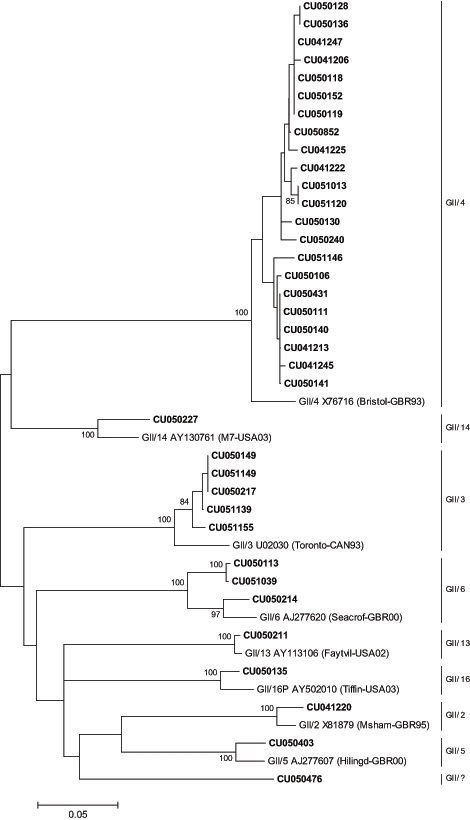
Phylogenetic analysis of norovirus genogroup II nucleotide sequences. The phylogenetic tree was generated by neighbor-joining analysis of the partial nucleotide sequences of the capsid N/S domain with bootstrapping of 1,000 replicates using MEGA version 3.1. Isolates identified in this study are highlighted in **boldface**. Bootstrap values >80 are shown at each node as percentage of 1,000 replicates. The scale bar represents the estimated number of substitutions per site. Nucleotide sequences obtained in this study were deposited in GenBank under accession nos. DQ522123–DQ522158. Nucleotide sequences of reference strains: Msham-GBR95 (GenBank accession no. X81879, genotype GII/2), Toronto-CAN93 (U02030, GII/3), Bristol-GBR93 (X76716, GII/4), Hilingd-GBR00 (AJ277607, GII/5), Seacrof-GBR00 (AJ277620, GII/6), Faytvil-USA02 (AY113106, GII/13), M7-USA03 (AY130761, GII/14), and Tiffin-USA03 (AY502010, GII/16).

Of the 63 NoV isolates, 43 (68.3%) were successfully sequenced for phylogenetic analysis, including 7 GI and 36 GII isolates. NoV GI isolates covered at least 5 genotypes, but no circulating strain predominated (Figure A2). For NoV GII isolates, we found >8 genotypes; GII/4 was the most prevalent (Figure A3).

To rule out the possibility of a quantitation artifact due to different stability between genogroups upon storage or freeze-thaw cycle, viral RNA was re-extracted and requantitated from a fecal specimen that had been stored for >6 months and coinfected with both NoV GI and GII. Repeat testing showed no drop in cDNA viral load for either genogroup, which suggests a comparable stability upon storage. Sequence complementarity among target, primers, and probe used in quantitation was also verified. While no sequence mismatch in primers and probe was found among the 7 NoV GI isolates, 5 of the 36 GII isolates had a single mismatch. Thus, the low cDNA viral load of NoV GI measured was unlikely due to sequence mismatching.

## Conclusions

In this study, we show for the first time that the median cDNA viral load of NoV GII is >100-fold higher than that of GI in the fecal specimens of patients with NoV-associated gastroenteritis. Neither the possibility of quantitation artifacts as a result of primers and probe mismatching nor stability differences between genogroups on storage was likely to account for our observation. Moreover, 7 of 9 specimens with NoV GI/GII coinfection exhibited higher cDNA viral load of GII than that of GI. Also, the cDNA viral load of NoV GII was usually higher than that of GI for each collection month, a finding that further supports our interpretation.

We speculate that the increased cDNA viral load facilitates the transmission of NoV GII from infected persons to susceptible hosts through the fecal-oral route. Studies on other viruses have shown that viral load correlates well with the epidemiology of diseases. For example, the predominance of HIV-1 over HIV-2 has been suggested to be attributed to the higher viral load of HIV-1 (*8*). However, the implication of viral shedding pattern and cDNA viral load on epidemiologic characteristics and clinical manifestations of NoVs deserves further investigation. Our findings provide strong molecular evidence for the worldwide predominance of NoV GII and may open new research directions in the epidemiologic study of NoVs.

## References

[1] Radford AD, Gaskell RM, Hart CA. Human norovirus infection and the lessons from animal caliciviruses. Curr Opin Infect Dis. 2004;17:471–8. 10.1097/00001432-200410000-00012 15353967

[2] Zheng DP, Ando T, Fankhauser RL, Beard RS, Glass RI, Monroe SS. Norovirus classification and proposed strain nomenclature. Virology. 2006;346:312–23. 10.1016/j.virol.2005.11.015 16343580

[3] Koopmans M, Strien EV, Vennema H. Molecular epidemiology of human caliciviruses. In: Desselberger U, Gray J, editors. Viral gastroenteritis. London: Elsevier; 2003. p. 523–54.

[4] Chan MC, Sung JJ, Lam RK, Chan PK, Lai RW, Leung WK. Sapovirus detection by quantitative real-time RT-PCR in clinical stool specimens. J Virol Methods. 2006;134:146–53. 10.1016/j.jviromet.2005.12.013 16427707

[5] Kageyama T, Kojima S, Shinohara M, Uchida K, Fukushi S, Hoshino FB, Broadly reactive and highly sensitive assay for Norwalk-like viruses based on real-time quantitative reverse transcription-PCR. J Clin Microbiol. 2003;41:1548–57. 10.1128/JCM.41.4.1548-1557.2003 12682144 PMC153860

[6] Pang XL, Lee B, Boroumand N, Leblanc B, Preiksaitis JK, Yu Ip CC. Increased detection of rotavirus using a real time reverse transcription-polymerase chain reaction (RT-PCR) assay in stool specimens from children with diarrhea. J Med Virol. 2004;72:496–501. 10.1002/jmv.20009 14748075

[7] Kageyama T, Shinohara M, Uchida K, Fukushi S, Hoshino FB, Kojima S, Coexistence of multiple genotypes, including newly identified genotypes, in outbreaks of gastroenteritis due to Norovirus in Japan. J Clin Microbiol. 2004;42:2988–95. 10.1128/JCM.42.7.2988-2995.2004 15243049 PMC446284

[8] De Cock KM, Adjorlolo G, Ekpini E, Sibailly T, Kouadio J, Maran M, Epidemiology and transmission of HIV-2. Why there is no HIV-2 pandemic. JAMA. 1993;270:2083–6. 10.1001/jama.1993.03510170073033 8147962

